# Characterization of the Active Constituents in Shixiao San Using Bioactivity Evaluation Followed by UPLC-QTOF and Markerlynx Analysis

**DOI:** 10.3390/molecules15096217

**Published:** 2010-09-03

**Authors:** Wei Zhou, Shu-Lan Su, Jin-Ao Duan, Jian-Ming Guo, Da-Wei Qian, Er-Xin Shang, Jin Zhang

**Affiliations:** Jiangsu Key laboratory for TCM Formulae Research (Supported by the Cyrus Tang Foundation of USA); Nanjing University of Chinese Medicine, Nanjing 210046, China; E-Mail: zhouwei19840@163.com (W.Z.)

**Keywords:** Shixiao San, analgesic activity, active components, UPLC-QTOF-MS, Markerlynx^TM^

## Abstract

Shixiao San is a famous Traditional Chinese Medicine (TCM) formula that has been used for a long time for the treatment of gynecological diseases. In this paper, the active constituents in Shixiao San were characterized by using bioactivity evaluation followed by UPLC-QTOF and Markerlynx^TM^ analysis. The analgesic activities of two extracts of Shixiao San were evaluated using the hot-plate test, acetic acid-induced writhing and dysmenorrhea mice model. The results showed that the analgesic activity of Shixiao San vinegary extract (boiling vinegar) was superior to the aqueous extract. UPLC-QTOF and Markerlynx^TM^ analytic results showed that the process of boiling in vinegar may improve the dissolution of Shixiao San flavanoids, and these flavanoids may contribute to the observed analgesic activity. This work demonstrated that UPLC/QTOFMS and Markerlynx^TM^ could serve as new methods for fast generation and automated analysis of information-rich data from Chinese herbal medicines.

## 1. Introduction

Shixiao San has analgesic activity and can dissipate blood stasis and it has been widely used in the clinic for centuries to treat gynecological disease [1]. Shixiao San consists of two crude herbs, Pollen Typha (Pollen of *Typha angustifolia* L.) named “Puhuang” in China, and Faeces Trogopterori (faeces of the complex-toothed flying squirrel *Trogopterus xanthipes* Milne-Edwards), named “Wulingzhi” in China*.*

It has been recorded in ancient documents like the *Complete Collection of Prescriptions for Women* that Shixiao San is first boiled with vinegar, and then extracted by water (Vinegary Extract). In addition, Shixiao San is clinically used after water extraction (Aqueous Extract) to treat many diseases [2]. The aqueous extract of Shixiao San has been reported to have multiple bio-activities, such as analgesia, sedation, anti-atherosclerotic, anti–inflammatory, and so on*.* [3,4]. The aqueous extract of Shixiao San was also reported to contain flavonoids, organic acids, esters of fatty acids, *etc.* [3,5], but until now the bioactivity and the chemical components of the vinegary extract of Shixiao San have not been reported. Moreover, there have been no studies of the differences in bio-activity and chemical components between the two Shixiao San preparations, therefore, in order to provide a scientific explanation and basis for clinical application, it is necessary to study and compare the bioactivity as well as the chemical constitution of the two extracts of Shixiao San.

It is well accepted that herbal medicines exert their efficacies on multi-targets through multi-components [6], and one or a few marker compound(s) cannot provide holistic information about a combinatorial formula [7]. However, due to the complexity of the chemical compositions of TCMs, the bioactive compounds and the therapeutic mechanisms of most TCMs are still unknown [8]. Therefore, new methods were needed to analyze the multi-components and their contribution to the efficacy of TCMs. 

Ultra performance liquid chromatography (UPLC) coupled with a photodiode array detector (PDA) and time-of-flight mass spectrometry (TOFMS) is a newly developed hyphenated technique (UPLC–PDA–TOFMS) [7]. It can provide a huge amount of information more rapidly and efficiently than other techniques. High selectivity and sensitivity have allowed the wide application of UPLC–MS/MS for quantitative and qualitative analysis, as well as metabolite analysis and identification from bioassays of complex samples such as TCMs [9]. To analyze and compare the information-rich spectroscopic data generated by UPLC/MS analysis from different samples, the commercial software Markerlynx^TM^ has been used to process the complex data quickly and reliably. MarkerLynx^TM^ is a peak detection algorithm, where each mass number is analyzed separately in a search for peaks. The area of these peaks will be given an identity of *m/z* and retention time and will be used as a fingerprint for each sample that is represented in relation to the other samples by principal component analysis. This software is repeatable and reliable analytical method when we should compare the information- rich spectroscopic data generated by UPLC/MS analysis from two or more group samples.

In this paper, the analgesic effects of two different extract of Shixiao San were investigated. Furthermore, UPLC-QTOF-MS coupled with Markerlynx^TM^ were used to analyze the chemical components in different extracts of Shixiao San. This work demonstrated the potential of the UPLC/QTOFMS approach using Markerlynx^TM^ combined with bioactivity evaluation for the characterization of the active constituents in Chinese herbal medicines

## 2. Results and Discussion

### 2.1. Effect of AESS and VESS on hot - plate test in mice

In the hot-plate test, the results presented in table 1 showed that AESS (Aqueous Extract of Shixiao San) did not prolong the latency time at the tested dosage. As to VESS (Vinegary Extract of Shixiao San), after 1 hour of administration, the latency time was significantly increased both at its high and low dosage compared with the control group (P < 0.05). 

**Table 1:** Effect of AESS and VESS on hot - plate test in mice (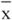 ± s, n = 10)^#^.

Group		Dosage (g/kg)	Hot - plate latency (s)	
			1 h	2 h
Control group		-	15.84 ± 3.81	15.40 ± 3.64
Pethidine group		0.025	32.37 ± 6.56^**^	16.45 ± 4.11
AESS group	High dose	4.90	19.71 ± 5.90	17.48 ± 6.74
	Low dose	2.45	19.40 ± 3.73	15.40 ± 3.66
VESS group	High dose	10.06	20.37 ± 3.56^*^	18.33 ± 4.53
	Low dose	5.03	20.21 ± 3.78^*^	18.13 ± 5.49

The crude extracts were ensured to be equal in dose converting into crude plant materials, and the doses of crude plant materials for two extracts are equal; # Values are expressed as mean ± standard deviations (n = 10); ** Level of significance relative to the control value P < 0.01; * Level of significance relative to the control value P < 0.05.

### 2.2. Effect of AESS and VESS on acetic acid - induced writhing movements in mice

Both AESS and VESS remarkably reduced the number of writhing times (P < 0.01) induced by acetic acid. VESS decreased the numbers of writhing to 32.70 ± 7.35 at its low dose, and the inhibition ratio was 47.71%, in comparison with that of AESS (20.50%) (Table 2).

**Table 2:** Effect of AESS and VESS on acetic acid - induced writhing movements in mice (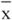 ± s, n = 10)^#^.

Group		Dosage (g/kg)	Number of writhings	Inhibition (%)
Control group		-	56.10 ± 5.86	-
Pethidine group		0.025	0.00 ^**^	100.00
AESS group	High dose	4.90	31.50 ± 5.19^**^	43.85
	Low dose	2.45	44.60 ± 12.06^*^	20.50
VESS group	High dose	10.06	29.11 ± 7.01^**^	48.10
	Low dose	5.03	32.70 ± 7.35^**^	47.71

The crude extracts were ensured to be equal in dose converting into crude plant materials, and the doses of crude plant materials for two extracts are equal; # Values are expressed as mean ± standard deviations (n = 10); ** Level of significance relative to the control value P < 0.01; * Level of significance relative to the control value P < 0.05.

### 2.3. Effects of AESS and VESS on dysmenorrhea mice models

All of the test medications could remarkably reduce the writhing times (P < 0.05) in a dymenorrhea mice model. The writhing inhibition rate of VESS low group is 60.68%, which is superior to AESS low group (39.25%) (as shown in Table 3). Meanwhile, the contents of Ca^2+^ and NO in the tissue homogenate of uterus were determined (Table 4). It was supposed that reduction of the writhing times by the tested medication was related to the decrease of Ca^2+^ and NO.

The previous three experiments showed that the bioactivity of VESS group was better than AESS group, which might be due to the chemical components changes during boiling with vinegar. So the chemical components in two different extracts are worthy of investigation.

**Table 3:** Effects of AESS and VESS on writhing of dysmenorrhea mice models (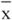 ± s, n = 10)^#^.

Group		Dose (g/kg)	Number of writhings	Inhibition (%)
Normal group		-	0	100
Control group		-	9.75 ± 3.86	-
Celecoxib group		0.0411	4.73 ± 3.87^*^	51.52
AESS group	High dose	4.90	5.00 ± 3.02^*^	48.71
	Low dose	2.45	5.92 ± 2.93^*^	39.25
VESS group	High dose	10.06	4.00 ± 2.66^*^	58.97
	Low dose	5.03	3.83 ± 2.66^*^	60.68

The crude extracts were ensured to be equal in dose converting into crude plant materials, and the doses of crude plant materials for two extracts are equal; # Values are expressed as mean ± standard deviations (n = 10); * Level of significance relative to the control value P < 0.05.

**Table 4:** Effects of AESS and VESS on the content of Ca^2+^ and NO in the uterus tissue homogenate (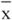 ± s, n = 10)^#^.

Group		Dose (g/kg)	Ca^2+^/ mmol·gprot^-1^	NO/ μmol·gprot^-1^
Normal group		-	0.08 ± 0.07	9.12 ± 3.24
Control group		-	0.44 ± 0.21	18.62 ± 4.12
Celecoxib group		0.0411	0.20 ± 0.14	18.73 ± 7.25
AESS group	High dose	4.90	0.13 ± 0.07^*^	9.15 ± 2.34^*^
	Low dose	2.45	0.22 ± 0.12^*^	11.98 ± 3.44^*^
VESS group	High dose	10.06	0.11 ± 0.18^*^	9.02 ± 2.95^*^
	Low dose	5.03	0.13 ± 0.11^*^	10.46 ± 3.05^*^

The crude extracts were ensured to be equal in dose converting into crude plant materials, and the doses of crude plant materials for two extracts are equal; # Values are expressed as mean ± standard deviations (n = 10); * Level of significance relative to the control value P < 0.05.

### 2.4. UPLC - QTOF - MS analytic results

**Figure 1:**
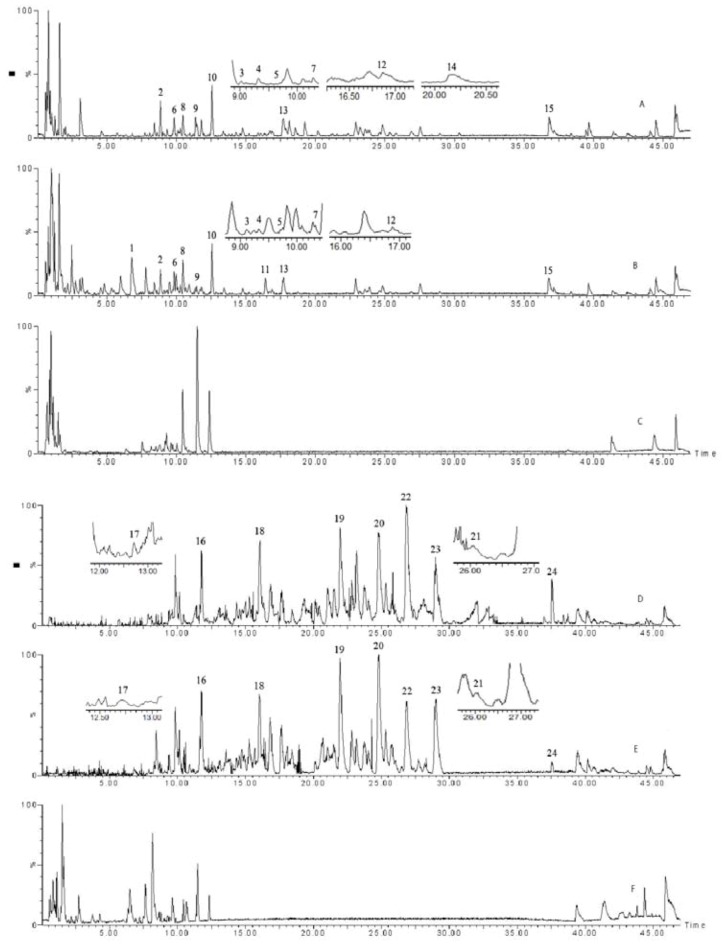
Total current chromatograms of AESS (A), VESS (B), Vinegar (C) in positive mode; and AESS (E), VESS (F), Vinegar (G) in negative mode.

Both the negative and positive ion modes were applied to analyze and identify the chemical components in the aqueous extract and vinegary extract of Shixiao San. The total current chromatograms with two ESI modes were shown in Figure 1. In order to eliminate the effects of vinegar, we analyzed the total ion chromatogram of vinegar in the same conditions. Twenty-four peaks were detected and eleven peaks were identified in two extracts, respectively. Through comparing the t_R_ values, UV λ_max_ values, and the MS characteristics of the peaks with reference compounds and the literatures, the eleven identified compounds are listed as follows: quercetin-3-*O*-(2G-α-l-rhamnosyl)-rutinoside, isorhamnetin-3-*O*-rutinoside, typhaneoside, isorhamnetin-3-*O*-neohesperidoside, kaempferol-3-*O*-neohesperidoside, quercetin-3,3′-dimethyl ether, quercetin-3-*O*-neohesperidoside, kaempferol-3-*O*-glucoside, afzelin, 2α,12β-dihydroxy-7,15-isopimardiene-18-oic-acid, and 2α,15S,16 -trihydroxy-8(14)-isopimarene-18-oic-acid. The information about the analyzed and identified compounds is summarized in Table 5.

**Table 5:** UPLC - MS analytic results of AESS and VESS.

NO.	Retention time (min)	Identification	UV λ _max_ (nm)	ESI^+^	ESI^-^	Source herb
1	6.79	unknown	225	-	231 [M+H]^+^, 253[M+Na]^+^	*FT*
2	8.83	unknown	228, 272	-	277 [M+H]^+^, 299 [M+Na]^+^	*PT/FT*
3	9.11	quercetin-3-*O*-(2G-α-l-rhamnosyl)-rutinoside	255, 353	755 [M−H]^−^, 300 [M−H−rha-glu-rha]^−^, 271 [M−2H−rha-glu-rha−CO]^−^, 255 [M−2H−rha-glu-rha−CO_2_]^−^	757 [M+H]^+^	*PT*
4	9.41	quercetin-3-*O*-neohesperidoside	255, 355	609 [M−H]^−^, 300 [M−H−rha-glu]^−^, 271 [M−2H−rha-glu−CO]^−^, 255 [M−2H− rha-glu−CO_2_]^−^	611 [M+H]^+^	*PT*
5	9.70	kaempferol-3-*O*-(2^G^-α-l-rhamnosyl)-rutinoside	265, 348	739 [M−H]^−^, 284 [M−H−rha-glu-rha]^−^, 255 [M−2H−rha-glu-rha−CO]^−^	741 [M+H]^+^, 763[M+Na]^+^	*PT*
6	9.81	isorhamnetin-3-*O*-(2^G^-α-l-rhamnosyl)-rutinoside	254, 354	769 [M−H]^−^, 314 [M−H−rha-glu-rha]^−^, 285 [M−2H−rha-glu-rha−CO]^−^	771 [M+H]^+^, 793[M+Na]^+^	*PT*
7	10.28	kaempferol-3-*O*-neohesperidoside	265, 348	593 [M−H]^−^, 284 [M−H−rha-glu-rha]^−^, 255 [M−2H−rha-glu-rha−CO]^−^	595 [M+H]^+^, 617 [M+Na]^+^	*PT*
8	10.47	isorhamnetin-3-*O*-neohesperidoside	254, 354	623 [M−H]^−^, 314 [M−H−rha-glu-rha]^−^, 285 [M−2H−rha-glu-rha−CO]^−^	625 [M+H]^+^, 647 [M+Na]^+^	*PT*
9	11.24	isorhamnetin-3-*O*-rutinoside	254, 354	623 [M−H]^−^, 314 [M−H−rha-glu-rha]^−^, 285 [M−2H−rha-glu-rha−CO]^−^	625 [M+H]^+^, 647 [M+Na]^+^	*PT*
10	12.55	unknow	-	-	679 [M+H]^+^, 701 [M+Na]^+^	*PT/FT*
11	16.42	unknow	-	-	621 [M+H]^+^, 643 [M+Na]^+^	*FT*
12	16.87	2α,12β-dihydroxy-7,15–isopimardiene–18–oic-acid	-	333 [M−H]^−^	335 [M+H]^+^	*FT*
13	17.71	unknow	-	-	621 [M+H]^+^	*FT*
14	20.27	2α,15S,16-trihydroxy-8(14)–isopimarene–18–oic-acid	-	351 [M−H]^−^, 387 [M+Cl]^−^	353 [M+H]^+^	*FT*
15	36.87	unknown	-	-	274 [M+H]^+^	*FT*
16	11.79	unknown	-	513 [M−H]^−^, 365, 315	-	*FT*
17	12.71	afzelin	264	431 [M−H]^−^	433 [M+H]^+^, 455 [M+Na]^+^	*FT*
18	16.05	unknown	-	349 [M−H]^−^	-	*FT*
19	22.00	unknown	-	331 [M−H]^−^, 119	-	*FT*
20	24.93	unknown	-	413 [M−H]−, 331, 119	-	*FT*
21	26.07	Quercetin-3,3′-dimethyl ether	254, 352	329 [M−H]^−^, 301[M−dimethyl ether]^−^	-	*PT*
22	26.87	unknown	-	329 [M−H]^−^, 215	-	*FT*
23	29.02	unknown	-	464 [M−H]−, 411, 329	-	*FT*
24	37.58	unknown	-	391 [M−H]−, 325, 243	-	*FT*

PT: Pollen typha; FT: Faeces trogopterori; - : not confirmed.

### 2.5. Markerlynx^TM^ analytic results

**Figure 2:**
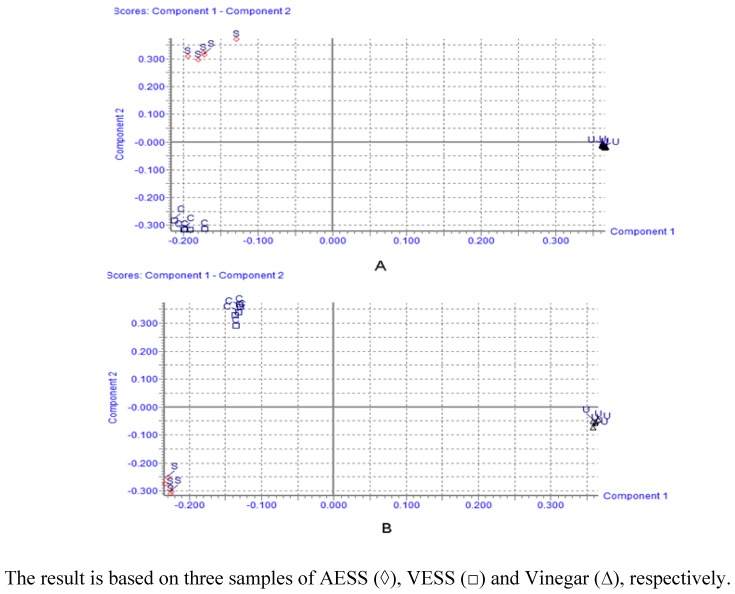
The scores plot obtained from Markerlynx^TM^ analytical results in positive mode (A) and negative mode (B).

**Figure 3:**
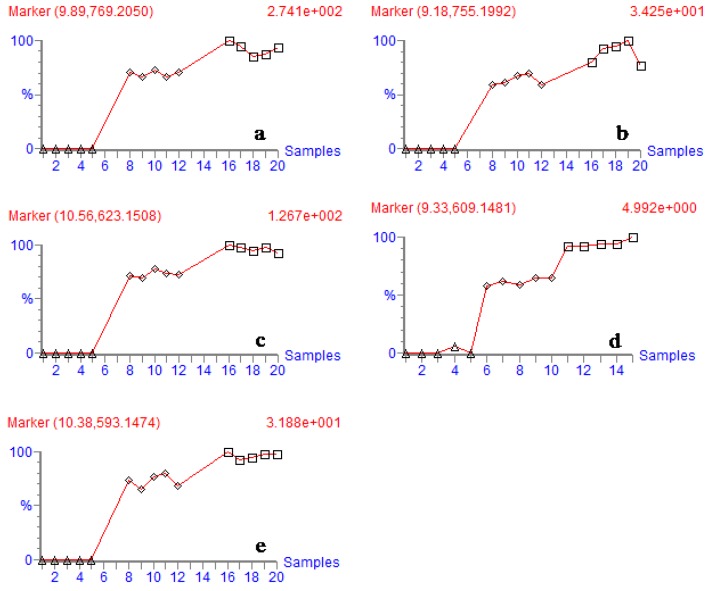
The variation trends of fragment ions intensity of identified compounds from three samples of AESS (◊) , VESS (□) and Vinegar (∆). (a) typhaneoside. (b) quercetin-3-*O*-(2G-α-L-rhamnosyl)-rutinoside. (c) isorhamnetin-3-*O*-neohesperidoside. (d) quercetin-3-*O*-neohesperidoside. (e) kaempferol-3-*O*-neohesperidoside.

The principal components analysis was done using the Waters Markerlynx^TM^ software. In this study, the sample of AESS, VESS and vinegar were injected five times each in two ESI modes. The results showed that the total current chromatograms of the three samples were significantly different, as shown in Figure 2. This can indicate that the chemical composition has changed after the process of boiling with vinegar. According to the intensity trends of fragment ions from three samples (Figure 3), the intensity of five flavonoids (typhaneoside, isorhamnetin-3-*O*-neohesperidoside, quercetin-3-*O*-neohesperidoside, kaempferol-3-*O*-neohesperidoside, quercetin-3-*O*-(2G-α-L-rhamnosyl)-rutinoside) were higher in VESS than of in AESS. Moreover, the above-mentioned compounds’ average values of peak area in VESS were list as follows: 238, 167, 123, 145, and 239. And the average values of peak area in AESS were 148, 115, 93, 103, and 174. So the contents of the detected five flavonoids were increased after the process of boiling in vinegar.

These trends can indicate the contents of the corresponding compounds in the three samples. The fragment ions here are the molecular ion (M^-^) peaks (x axis: the number of sample injection; y axis: fragment ions abundances).

### 2.6. Discussion

In this work, we first developed a new method of using bioactivity evaluation followed by UPLC/QTOFMS and Markerlynx^TM^ to quickly determine and analyze the information-rich data for characterizing the active components in TCMs. Moreover, this work demonstrated that this potential approach for the characterization of the active constituents in TCMs is effective and feasible.

It was considered in Traditional Chinese Medicine that the TCM formula involves the compatibility of two or more medications, that could interact and increase the effect of any single drug. In the present study, the compatibility of two medications (Puhuang - Wulingzhi) resulted in remarkable biological effects. After the process of boiling with vinegar, the effects were promoted. The results in this paper suggested that the process of boiling in vinegar might improve dissolution of active components and enhance the analgesic activity.

The hot-plate test and acetic acid-induced writhing test reflected central and peripheral nociceptive model respectively. Dysmenorrhea mice model were adopted to evaluate the analgesic activity of Shixiao San. The results showed that the analgesic effects of vinegary extract were better than those of the aqueous extract. Shixiao San is frequently used to treat primary dysmenorrheal in the clinic in China [2]. The mechanism of primary dysmenorrhea may be related to intracellular free calcium and the levels of prostaglandin F_2__α_. In addition, NO is the active factor involved in the stimulation of COX-2 enzyme [10,11,12]. This study showed that Shixiao San extracts significantly inhibited the writhing response on the primary dysmenorrhea mice, and reduced the content of Ca^2+^ and NO in uterine tissue. Therefore these data implied that the mechanisms of inhibitory activity on myometrial contractility of VESS or AESS were related with the lower of Ca^2+^ and NO contents in the smooth muscle cell.

It was known that multiple constituents are responsible for their bioactivities of TCMs [13], however, due to the complexity of chemical compositions, the responsible bioactive compounds are difficult to confirm. Therefore, it is important and urgent to carry out chemical and pharmacological correlated studies on TCMs to elucidate the biologically active compounds and their therapeutic mechanisms [14]. In this paper, the method combining pharmacological experiments and UPLC/QTOFMS and Markerlynx^TM^ proved effective for the fast characterization of the active constituents in different preparations of Shixiao San. This method is a simple, repeatable and reliable testing technique for comparing the information-rich spectroscopic data generated by UPLC/MS analysis from two or more group samples. It can reveal the difference of various samples much more quickly and exactly than traditional approaches like HPLC-DAD.

The data of chemical constituents’ changes between aqueous extract and vinegar extract showed that the total current chromatograms of two samples were different significantly, and the ion intensity of five flavonoid compounds were also different through the UPLC/QTOFMS coupled with Markerlynx^TM^ method. It can be inferred that the processing of vinegar may promote the dissolution of flavonoids by intermolecular hydrogen bonds, so the changing components in different extracts may result in different bioactivities, and these components (flavones glycosides) may be the active constituents. Moreover, these flavonoids possessed multiple activities as antiflammatory, analgesic, immunosuppressive compounds [15,16,17]. Modern pharmacological studies show that kaempferol-3-*O*-neohesperidoside and quercetin-3-*O*-neohesperidoside can inhibit the activity of nitric oxide produced by activated macrophages [18] and kaempferol glycosides may account for the renowned medicinal use of Sedum dendroideum against pain and inflammatory problems [19].

## 3. Experimental

### 3.1. Materials

The raw materials of Pollen Typha and Faeces Trogopterori were purchased from Jiansu (Yixing) and Hebei (Anguo), respectively. All the crude herbs were identified by the corresponding author. Voucher specimens (No. NJUTCM - 20090118 ~ 20090119) were deposited in Jiangsu Key Laboratory for TCM Formulae Research, Nanjing University of Chinese Medicine, China.

### 3.2. Chemicals and reagents

Estradiol benzoate injection (Tianjin Jinyao Amino Acid Pharmaceutical Co., Ltd., Tianjin, China NO. 0705161), oxytocin injection (Shanghai First Biochemical Pharmaceutical Co., Ltd., Shanghai, China, NO. 071116), Celecoxib capsules (Pfizer Pharmaceuticals LLC, NO. BK081004), Ca^2+^ test kit and NO test kit (Nanjing Jiancheng Bioengineering Institute Co., Ltd., Nanjing, China, NO. 20081204; NO. 20081204), pethidine injection (Qinghai Pharmaceutical Co., Ltd., Qinghai, China, No. 050310), Vinegar (Jiangsu Hengshun Vinegar Co., Ltd., Jiangsu, Zhenjiang, China).

Acetonitrile was HPLC-grade from Merck (Darmstadt, Germany) and deionized water was purified by an EPED super purification system (Eped, Nanjing, China). The reference compounds typhaneoside, isohamnetin-3-*O*-neohespeidoside, and isohamnetin-3-*O*-rutinoside were purchased from the National Institute for the Control of Pharmaceutical and Biological Products (Beijing, China), quercetin-3-*O*-(2G-α-L–rhamnosyl)-rutinoside, afzelin, kaempferol-3-*O*-glucoside, kaempferol-3-*O*-neohesperidoside, quercetin-3,3′-dimethyl ether, quercetin-3-*O*-neohesperidoside, 2,12-dihydroxy-7,15-isopimardiene-18-oic-acid and 2,15S,16- trihydroxy-8(14)-isopimarene-18-oic-acid were isolated by our laboratory. Other reagents solutions were analytical grade (Sinopharm Chemical Reagent Co., Ltd., Shanghai, China). 

### 3.3. Animals

All experiments were performed with female ICR mice, weighing 18–22 g, obtained from the experimental animal center of China Pharmaceutical University. They were kept in plastic cages at 22 ± 2 ºC with free access to pellet food and water and on a 12 h light/dark cycle. Animal welfare and experimental procedures were carried out in accordance with the guide for the care and use of laboratory animals (National Research Council of USA, 1996) and related ethical regulations of our university. Groups each with 10 animals were used in all tests.

### 3.4. Instrumentation

Chromatographic experiments were performed on a Waters ACQUITY™ UPLC™ system connected to Synapt™ Q-TOF Premier equipped with an electrospray ionization source, which was used in positive or negative mode(Waters Corp., Milford, MA, USA). UPLC separation was achieved using an ACQUITY UPLC™ BEH C_18_ column (100 × 2.1 mm, 1.7 μm). Data acquisition and processing were performed using MassLynx 4.1 and Markerlynx 4.1 (Waters Corp., Milford, MA, USA). MarkerLynx^TM^ Application Manager is a software package from Waters (Mass., USA) for peak detection. Automatic Microplate Reader (Thermo, USA); TGL - 16C High Speed Centrifuge (Shanghai Anting Scientific Device Co., Ltd., Shanghai, China); AL204 Electronic Balance (Mettler Toledo); YLS - 6B Hot Plate (Beijing Gene & I Scientific Ltd., Beijing, China). 

### 3.5. Preparation of samples

#### 3.5.1. Preparation of aqueous extract of Shixiao San (AESS)

Puhuang and Wulingzhi (500 g respectively) were extracted twice (1 h/time) under reflux with 10,000 mL water (pH = 7.5). The combined extract was concentrated under reduced pressure to give the dried extract (yield 11.9%) as the extract sample 1.

#### 3.5.2. Preparation of vinegary extract of Shixiao San (VESS)

Puhuang and Wulingzhi (500 g respectively) were boiled with 1,000 mL vinegar (pH = 3.5) for about 2.5 h until dryness, and then extracted twice (1 h/time) under reflux with 10,000 mL water. The combined extract was concentrated under reduced pressure to give the dried extract (yield 25.7%) as the extract sample 2.

### 3.6. Hot - plate test in mice

Experiments were carried out according to the previously described method [20]. Mice were pretreated with test medication, respectively. Animals were habituated twice to the hot-plate in advance. For testing, mice were placed on hot-plate maintained at 55 ± 5 ºC. The interval of time between occurrence of either a hind paw licking or a jump off the surface was recorded as the hot- plate latency. Mice with baseline latencies of < 5 s or > 30 s were eliminated from the study. Hot-plate latencies were determined at 60 min and 120 min after oral administration of the tested medication. 

ICR mice were divided into six groups, and the control group was administrated isovolumetric distilled water. The typical dose of crude plant material of Shixiao San for a person was about 20 g/day (Puhuang and Wulingzhi was 10 g respectively) according to folk remedies. After the preliminary experiments, two effective dosages were selected, and the crude extracts were adjusted to be equal in dose converting into crude plant materials. The dosage of the test medication was AESS group i. g. 4.9 g/kg, and 2.45 g/kg of crude extracts, VESS group i. g. 10.06 g/kg, and 5.03 g/kg of crude extracts, and the mice were administered for seven days. The mice of the positive group were injected pethidine before being placed on the hot-plate.

### 3.7. Acetic acid - induced writhing response in mice

The acetic acid - induced writhing model was basically established as described in the literatures [21,22]. ICR mice were pretreated with test medication for 7 days, 1 h before the injection of 0.6% aqueous solution of acetic acid (10 mL/kg, i. p.) last time. Each mouse was placed in a transparent observation box and the number of writhings was counted for 20 min after the acetic acid administration. The number of writhes in each treated group was compared with the control group. The inhibition rate of writhing [(control mean − test mean) / control mean] × 100 was calculated. ICR mice were divided into 6six groups, and control group was administrated isovolumetric distilled water. The dosage of the test medication was the same as previous experiments, and the mice were administered for seven days. The mice of positive group were injected pethidine.

### 3.8. Dysmenorrhea mice model preparation

According to the reported method [23,24], we used estradiol benzoate and oxytocin to make the dysmenorrhea mice model. Estradiol benzoate (0.01 g/kg) was administrated by subcutaneous injection for 6 days, and on the seventh day, oxytocin (10 mL/kg) was administrated by peritoneal injection 60 min after the final administration. ICR mice were divided into six groups, and the normal group was administrated isovolumetric distilled water, the control group was treated with estradiol benzoate and oxytocin and administrated distilled water. The dosage of the test medication was the same as previous experiments. The celecoxib group which was positive group was treated with estradiol benzoate and oxytocin and i. g. 0.0411 g/kg of what. From the fifth day, all the test medications were administrated for three days. Record the number of writhings occurring from 5 to 20 min, and determine the content of Ca^2+^ and NO in the homogenate of uterus according to specification of kits.

### 3.9. Statistical analysis

The results were expressed as mean ± S.D. and evaluated with one - way ANOVA following by Dunnett’s multiple comparisons test. A *p* value of less than 0.05 was considered statistically significant and *p* less than 0.01 being very significant.

### 3.10. UPLC – QTOF - MS analytical conditions

The mobile phase was composed of A (acetonitrile), B (0.5% aqueous acetic acid, v/v) with a linear gradient elution: 0~5min, A: 5%; 5~8 min, A: 5%~15%; 8~11 min, A: 15%~20%; 11~19 min, A: 20%~25%; 19~35 min, A: 25%~35%; 35~43 min, A: 35%~80%; 43~45 min, A: 80%; 45~46 min, A: 80 %~5 %; 46~47min, A: 5%. The flow rate of the mobile phase was 0.4 mL/min, and the column temperature was maintained at 30 ºC. The ionization source conditions were as follows: capillary voltage of 1.7 kV, source temperature of 100 ºC and desolvation temperature of 250 ºC. The sampling cone voltage was set at 40 V, extraction cone at 0.8 V, trap collision energy 6.0, transfer collision energy 4.0, trap gas flow were 1.50 mL/min, ion energy at 1.0 V, collision energy at 4.0 V. Nitrogen and argon were used as cone and collision gases, respectively. The cone and desolvation gas flow were 50 and 600 L/h, respectively. The scan time of 0.6 s and with interval scan time of 0.02 s was used throughout and with collision energy of 6 eV. The mass spectrometric data was collected from *m/z* 100 to 1000 in positive and negative ion in centroid mode. The MS/MS experiments were performed at variable collision energies (6–40 eV) which were optimized for each individual compound.

### 3.11. MarkerLynx^TM^

Data processing were performed using Markerlynx^TM^ software which with the help of multivariate statistical analysis, revealed the possible changed components, and the identities of which can be extensively determined with online UV and MS information. MarkerLynx^TM^ operates in two steps: firstly, peak detection is performed using ApexPeak Track. The peaks from different samples were aligned so that the same peaks (RT, *m/z*), *i.e*., most probably the same compound, were found in the same row for all samples. In this process, sample name and ion intensity were analyzed by supervised orthogonal partial least squared discriminate analysis (OPLS–DA) using the MarkerLynx^TM^ software; and then, all of the results, including the Markers table and PCA Scores and loading plots are viewed via the MarkerLynx browser. The ion intensities for each detected peak were normalized against the sum of the peak intensities within that sample using MarkerLynx^TM^. Ions of different samples were considered to be the same ion when they demonstrated the same t_R_ (tolerance of 0.01 min) and m/s value (tolerance of 0.01 Da). If a peak was not detected in a sample, the ion intensity was documented as zero in the table.

The original data were processed using the following parameters: initial retention time 0 min, final retention time 40 min, mass tolerance 0.02 Da, mass window 0.02 Da, retention time window 0.1 min, noise elimination level 6. PCA reduces a complex data set to a 2D scores plot showing the intrinsic patterns in the data and a 2D loading plot that highlights the quantities responsible for the patterns.

## 4. Conclusion

In this paper, the results proved that the components changes of Shixiao San triggered by different preparations generated different bioactivities. The advantages of the proposed method over the conventional chromatographic fingerprints and marker compounds are the speed and reliability to determine the potential active components in different extracts, such as VESS and AESS. The data in this article suggested that the new method could provide thoughts and techniques for revealing the possible interactions between TCMs.
